# Exponential-Distance Weights for Reducing Grid-like Artifacts in Patch-Based Medical Image Registration

**DOI:** 10.3390/s21217112

**Published:** 2021-10-26

**Authors:** Liang Wu, Shunbo Hu, Changchun Liu

**Affiliations:** 1 School of Control Science and Engineering, Shandong University, Jinan 250061, China; wuliang@mail.sdu.edu.cn; 2 School of Information Science and Engineering, Linyi University, Linyi 276005, China

**Keywords:** patch-based, registration, overlap, distance, exponential function

## Abstract

Patch-based medical image registration has been well explored in recent decades. However, the patch fusion process can generate grid-like artifacts along the edge of patches for the following two reasons: firstly, in order to ensure the same size of input and output, zero-padding is used, which causes uncertainty in the edges of the output feature map during the feature extraction process; secondly, the sliding window extraction patch with different strides will result in different degrees of grid-like artifacts. In this paper, we propose an exponential-distance-weighted (EDW) method to remove grid-like artifacts. To consider the uncertainty of predictions near patch edges, we used an exponential function to convert the distance from the point in the overlapping regions to the center point of the patch into a weighting coefficient. This gave lower weights to areas near the patch edges, to decrease the uncertainty predictions. Finally, the dense displacement field was obtained by this EDW weighting method. We used the OASIS-3 dataset to evaluate the performance of our method. The experimental results show that the proposed EDW patch fusion method removed grid-like artifacts and improved the dice similarity coefficient superior to those of several state-of-the-art methods. The proposed fusion method can be used together with any patch-based registration model.

## 1. Introduction

Medical image registration aims to generate a dense displacement field (DDF) to accurately register a pair of images, and to spatially align anatomical structures [1]. It is a fundamental procedure in various medical image analysis tasks [2]. However, to find the best DDF requires many iterative optimizations between the two images, and the traditional algorithm that is used has a higher time cost [3].

With the rapid development of deep learning, learning-based medical image registration methods have been commonly applied in recent years. It can imitate the process of traditional image registration methods and quickly predict the DDF of two unseen images by training a deep neural network. Patch-based training is not affected by shortage of training datasets as much, since many image patches can be sampled from the original images. In addition, patch-based training usually has better performance locally than whole-image-based training [4]. One challenge regarding patch-based image registration is the patch fusion process, which stacks many image patches to generate the final whole-image transformation. This patch fusion process can generate grid-like artifacts along the edges of the patches. To solve this problem, Yang et al. [5] introduced Quicksilver, a fast-deformable image registration method. During inference, they provided a probabilistic version which can calculate uncertainties in the predicted deformations. Cao et al. [6] proposed a novel deformable registration method, which is based on a cue-aware deep regression network. In the application stage, the first step was to extract patches from image pairs using a key-point sampling strategy. Then, the DDF patch was predicted by the deep regression network. Finally, the whole DDF could be obtained by the block-wise thin-plate spline (TPS) interpolation. Fan et al. [7] introduced a dual-guided, fully convolutional network for brain image registration, which estimates only the DDF in the central region. The size of the input image pair was 64 × 64 × 64 voxels, and the output was a 24 × 24 × 24 DDF patch. Hu et al. [8] used a stride of four to generate patches which can produce a smoother DDF in the inference. At the same time, to further increase the smoothness of the DDF and to ensure that it contained enough neighborhood information, surface-discarding was adopted. Finally, the whole DDF was obtained by the arithmetic average weighted (AAW) method.

Most of these methods can remove grid-like artifacts of DDFs with probabilistic models, a small stride, and estimating only the central region, etc. However, they cannot quickly obtain DDF without these grid-like artifacts at a large stride. Hence, we have applied an exponential-distance-weighted (EDW) scheme to the patch fusion process. Compared with the fusion methods used in the above literature, the EDW patch fusion strategy proposed in this paper can achieve more significant performance in a larger stride in a shorter time. Our code is freely available at https://github.com/LiangWUSDU/EDW (accessed on 24 September 2021). The main contributions are summarized as follows:

1. We calculate the relative weight of each point prediction using an exponential function on each patch, according to the distance from each point to the center point. This allows fusion of the predictions from all overlapping patches, while giving lower weight to predictions that are made by the patches near their edges.
2. The proposed patch fusion method can be used together with a patch-based deep learning model for registration without any modification to significantly improve network predictions.

## 2. Methods

### 2.1. Grid-like Artifacts

There are two main reasons for the emergence of grid-like artifacts: (1) the influence of using zero-padding in the feature extraction process. (2) the influence of strides on the choosing of patches in the testing stage.

**Figure 1:**
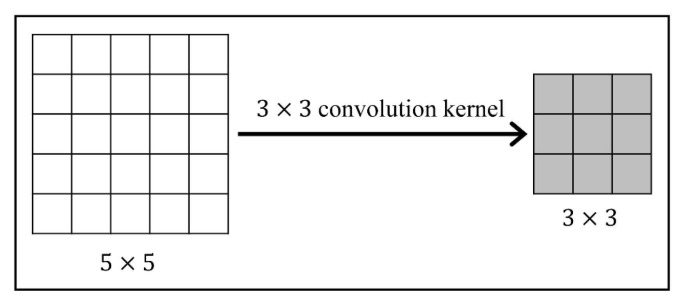
Illustration of Convolution.

**Figure 2:**
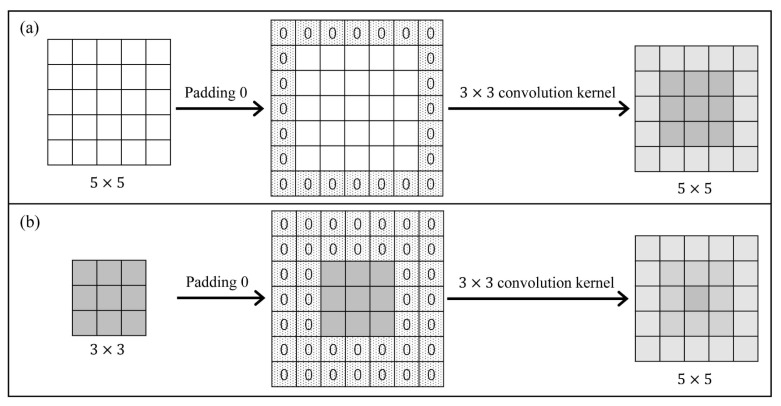
Illustration of (**a**) Zero Padding and (**b**) Transposed Convolution.

Deep neural networks (DNNs) have been successfully applied in order to enhance the state-of-the-art of many segmentation, registration and classification tasks. Among them, convolution is often used as an effective method for feature extraction. By sliding the filter over the input feature maps, the dot product is taken between the filter and the parts of the input feature. The output is a new set of feature maps. As shown in Figure 1, in 2D convolution, the stride is 1, the input is a 5 × 5 feature map, and the output is a 3 × 3 feature map, by a 3 × 3 convolution kernel. In some image processing tasks such as image registration and segmentation, the output and input should have the same size. Zero-padding and transposed convolution are the two most popular approaches. As shown in Figure 2a, zero-padding allow us to control the size of the feature map, by padding 0 to make the output size the same as the input size. In Figure 2b, transposed convolution is the reverse of normal convolution, but only regarding size. To ensure the same size of the output as the input in this convolution operation, it is necessary to perform padding 0 before convolution. This kind of zero-padding increases the uncertainty of the edge regions. The same conclusion is obtained for the transposed convolution.

**Figure 3:**
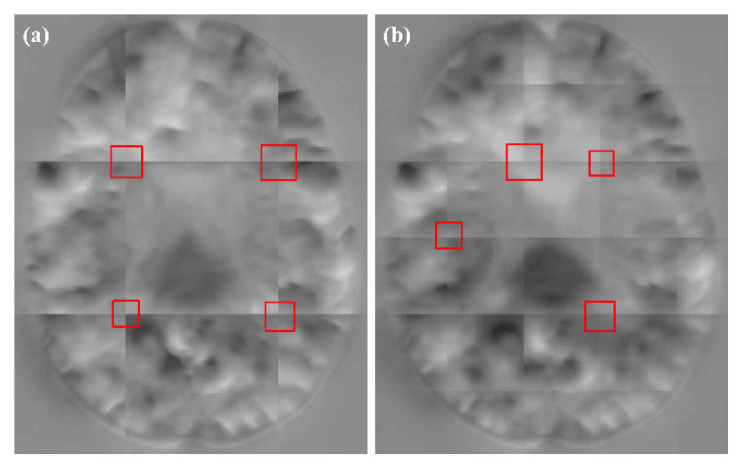
DDF obtained by fusion (**a**) without patch overlapping and (**b**) patch overlapping.

In the patch-based image registration method, due to the image features in the patch edges being incomplete, the patch prediction at the patch edges is less accurate. In addition, the extraction of patches using sliding windows with different strides also produces different degrees of grid-like artifacts. In Figure 3a, when the stride is consistent with the patch size, there is no overlap between the patches, which leads to severe grid-like artifacts after fusion, due to the inconsistent information of each patch edge. When the stride is smaller than the patch size, the AAW is generally used for the overlapping regions. The weights of the overlapping regions are the same no matter how far away from the center they are. The same grid-like artifacts will appear, as shown in Figure 3b. In the patch-based training method, the central region is more informative than the edge region. It is not reasonable to use the same weight for the edge region and the central region. Therefore, this study set the weight using an exponential function which was calculated by the distance from the points in the patch to the center point.

### 2.2. Distance Functions

Some of the common distance functions in the field of image processing are Euclidean distance [9], Manhattan distance [9], Chebyshev distance [10] et al. These three functions are all metrics which compute a distance value based on two data points, and they are widely used in medical image processing [11,12]. Hence, in this work, we evaluated these three distance functions. The patch size is h×w×c. In the patch coordinate system, the coordinate of any point is $\left(i_{P},j_{P},k_{P}\right)$, $i_{P}\in [0,h-1]$, $j_{P}\in [0,w-1]$, $k_{P}\in [0,c-1]$. The coordinate of the center point of the patch is $\left(\frac{h-1}{2},\frac{w-1}{2},\frac{c-1}{2}\right)$.

#### 2.2.1. Euclidean Distance

Euclidean distance is the most widely used distance metric. The Euclidean distance $d_{e}$, between two points in Euclidean space, is the length of the line segment between the two points, as:

(1) $$d_{e}=\sqrt{{\left(i_{P}-\frac{h-1}{2}\right)}^{2}+{\left(j_{P}-\frac{w-1}{2}\right)}^{2}+{\left(k_{P}-\frac{c-1}{2}\right)}^{2}}$$

#### 2.2.2. Manhattan Distance

Manhattan distance is calculated as the sum of the absolute differences between the two points. The Manhattan distance $d_{m}$ of two points is:

(2) $$d_{m}=\left|i_{P}-\frac{h-1}{2}\right|+\left|j_{P}-\frac{w-1}{2}\right|+\left|k_{P}-\frac{c-1}{2}\right|$$

#### 2.2.3. Chebyshev Distance

The Chebyshev distance between two points is the maximum absolute magnitude of the differences between the coordinates of the points. The Chebyshev distance $d_{c}$ of two points is:

(3) $$d_{c}=\max(\left|i_{P}-\frac{h-1}{2}\right|,\left|j_{P}-\frac{w-1}{2}\right|,\left|k_{P}-\frac{c-1}{2}\right|)$$

**Figure 4:**
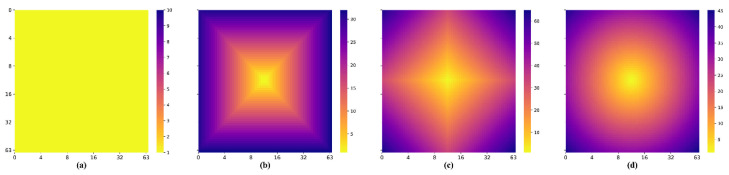
The heat map of the weights used by the AAW method, and the distances obtained by using different distance functions in the 33rd slice of a 64 × 64 × 64 voxels patch. (**a**) AAW method; (**b**) Chebyshev distance; (**c**) Manhattan distance; (**d**) Euclidean distance.

**Figure 5:**
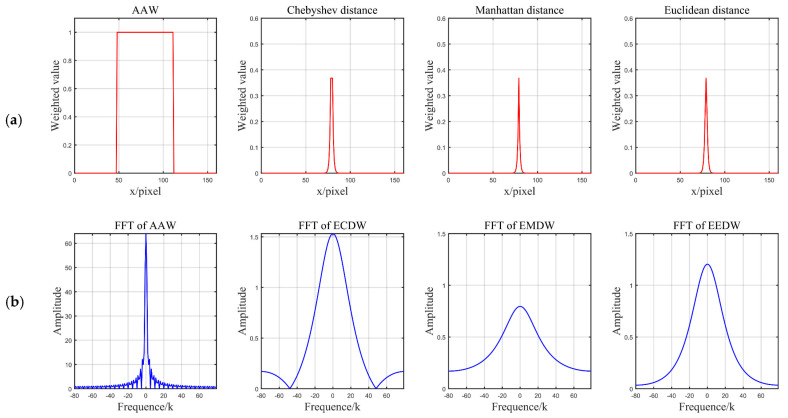
Plot of the weights of the AAW method, the exponential weighted distance (**a**) and the magnitude of the corresponding Fourier transformation (**b**) for the 32nd row of the 33rd slice. From left to right: AAW, exponential Chebyshev-distance-weighted (ECDW), exponential Manhattan-distance-weighted (EMDW), and exponential Euclidean-distance-weighted (EEDW). From the amplitude of FFT, we can find that the AAW method and ECDW retain some high-frequency information and cannot remove the grid-like artifacts to a greater degree.

In Figure 4b–d, the deep blue color indicates a large distance from the center point, and the deep yellow color indicates a small distance. It can be visualized that all three distance functions become smaller when they are closer to the center point. In addition, they increase from the center point to the surrounding area in a square, star, and circle manner, respectively. Additionally, in the heat map of the AAW method, the colors are the same for all positions, indicating that each position receives the same weight. From the frequency domain analysis (Figure 5), the amplitude of the AAW method oscillates, indicating that it still had more high-frequency components. From the amplitude of the Chebyshev distance function, we observed that it also retained a small number of high-frequency components. Therefore, these two methods would still have grid-like artifacts on DDFs. The amplitude of Manhattan distance and Euclidean distance are relatively stable, which can achieve the purpose of removing grid-like artifacts while preserving the original information.

### 2.3. The Process of Patch Fusion

**Figure 6:**
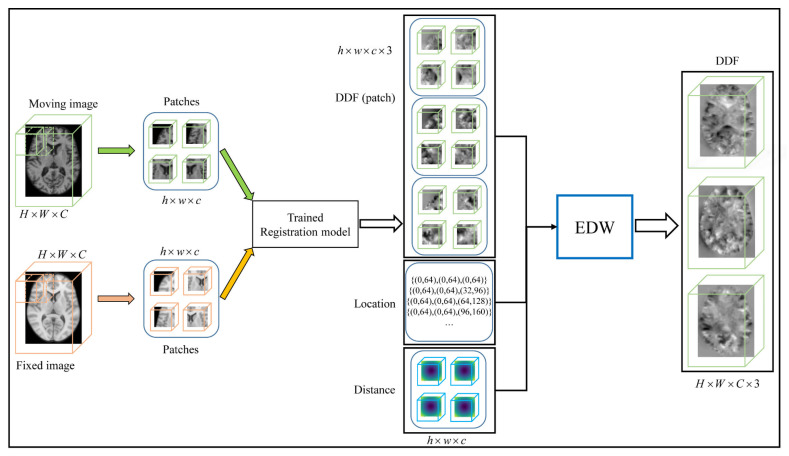
The pipeline of patch fusion in the test phase. The size of both the moving image and the fixed image is H×W×C, and the overlapping patches of size h×w×c are extracted using the sliding window with stride s. The DDF patch of h×w×c×3 is obtained by the trained registration model. The output DDF patch is located at the same location as the input patch. Finally, the whole DDF is obtained by the EDW method.

In our work (as illustrated in Figure 6), we extract overlapping patches via a sliding window with a stride of s.

An MR image can generate m patches, which can be calculated as follows:

(4) $$m=\left\lfloor \frac{H-(h-s)}{s}\right\rfloor \left\lfloor \frac{W-(w-s)}{s}\right\rfloor \left\lfloor \frac{C-(c-s)}{s}\right\rfloor$$

where, H×W×C is the image size, and h×w×c is the patch size. During inference, the output DDF patch is of a size h×w×c×3. The prediction values of each position are $\phi^{i,j,k}=\{\phi_{1}^{i,j,k},\phi_{2}^{i,j,k},\ldots ,\phi_{n}^{i,j,k}\}$, and n is the number of overlaps at point (i,j,k), where $n=n_{i}\times n_{j}\times n_{k}$, i∈[0,H−1], j∈[0,W−1], k∈[0,C−1]. $n_{i}$ can be calculated as follows:

(5) $$n_{i}=\{\begin{array}{cc} \left\lfloor \frac{i}{s}\right\rfloor +1, & i<h\\ \frac{h}{s}, & h\leq i\leq \left\lfloor \frac{H-(h-s)}{s}\right\rfloor s-1\\ \left\lfloor \frac{(\left\lfloor \frac{H+s}{s}\right\rfloor -1)s-i}{s}\right\rfloor , & \left\lfloor \frac{H-(h-s)}{s}\right\rfloor s-1<i<(\left\lfloor \frac{H+s}{s}\right\rfloor -1)s\\ 0, & (\left\lfloor \frac{H+s}{s}\right\rfloor -1)s\leq i \end{array}$$

**Figure 7:**
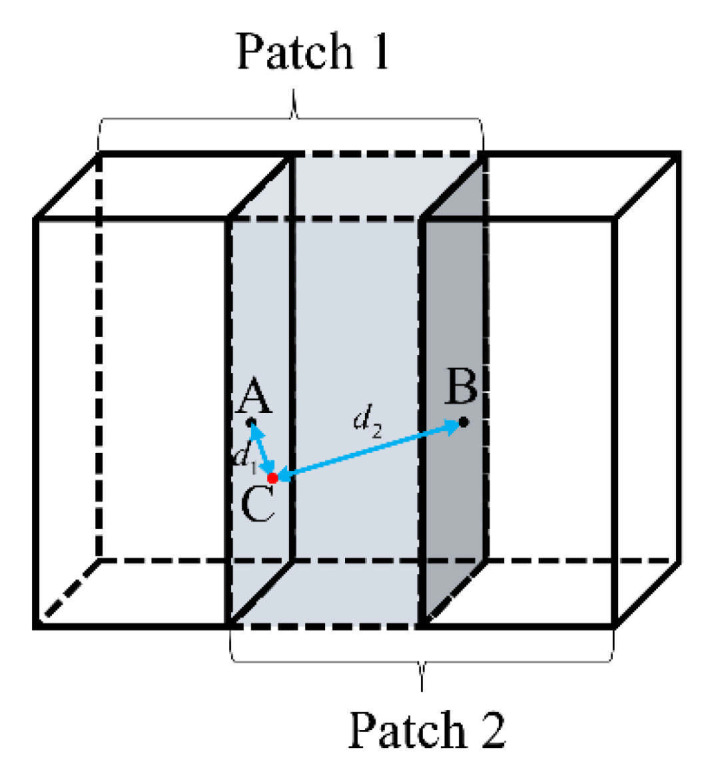
A schematic diagram of the overlapping patches. The shaded regions represent overlap. Points A and B are the center points of patch 1 and patch 2, respectively, and point C is a point in the overlap region. The predicted value of point C in patch 1 and patch 2 are $\phi_{A}$ and $\phi_{B}$, respectively. $d_{1}$ is the distance from point C to point A when point C is in patch 1, $d_{2}$ is the distance from point C to point B when point C is in patch 2. The final predicted value is $\phi_{C}$. According to Equation (6), the weights of point C in patch 1 and patch 2 are as follows: $\omega_{A}=\frac{e^{-d_{1}}}{e^{-d_{1}}+e^{-d_{2}}}$ and $\omega_{B}=\frac{e^{-d_{2}}}{e^{-d_{1}}+e^{-d_{2}}}$. Finally, we can get $\phi_{C}=\omega_{A}\phi_{A}+\omega_{B}\phi_{B}$ from Equation (7).

We can calculate $n_{j}$,$n_{k}$ by the same way. According to the distance map of each patch, we can determine the corresponding distance $d^{i,j,k}=\{d_{1}^{i,j,k},d_{2}^{i,j,k},\ldots ,d_{n}^{i,j,k}\}$ for n overlapping patches, at position (i,j,k). Due to the uncertainty of the edges, we use the following equation to obtain the normalized weights:

(6) $$\omega_{ts}^{i,j,k}=\frac{e^{-d_{ts}^{i,j,k}}}{\sum_{t=1}^{n}e^{-d_{ts}^{i,j,k}}}$$

where, $\omega_{ts}^{i,j,k}$ is the weight of t overlapping patches at the point (i,j,k), t∈[1,n], n is the number of overlapping points (i,j,k) when extracting the patch with a stride of s. *e* (≈2.71828), a natural constant which is the base of the exponential function. From Figure 7 we observed that the further the distance from the patched centroid is, the smaller the value of the weight is. This can give a greater confidence to the central region and improve the final prediction. Hence, the final prediction value of this point is:

(7) $${\hat{\phi }}_{s}^{i,j,k}=\sum_{t=1}^{n}\omega_{ts}^{i,j,k}\phi_{ts}^{i,j,k}$$

where, ${\hat{\phi }}_{s}^{i,j,k}$ is the final predicted value of the DDF at point (i,j,k). The whole DDF $\hat{\phi }$ can be obtained by applying the above processing to all voxels.

## 3. Experiments and Results

### 3.1. Dataset Description

Experimental data were obtained from The Open Access Series of Imaging Studies (OAISIS-3) (https://www.oasis-brains.org, accessed on 15 July 2021) [13]. The OASIS-3 dataset was a longitudinal imaging, clinical and cognitive dataset of both normal aging and Alzheimer’s disease. It included 609 cognitively normal subjects and 489 subjects with varying stages of cognitive decline. We randomly selected 800 TI-weighted (T1) subjects for the experiment. We used 750 subjects for training, 50 subjects for inference, and the MNI-152 brain atlas was used as the fixed image. In preprocessing, each subject was linearly aligned to the MNI-152 brain atlas. The final image size was 160 × 192 × 160 voxels with 1 × 1 × 1 mm^3^ voxel resolution. The dataset also contained segmentation label images of cerebrospinal fluid (CSF), gray matter (GM), and white matter (WM).

### 3.2. Experimental Details

During training, we extracted 80 patches with sizes 64 × 64 × 64 from each image with a stride of 32. The total number of patch pairs of MR images was 600,000. During reference, we explored the performance of the proposed fusion method by extracting the patches using sliding windows with different strides according to Equation (4), as shown in Table 1. We evaluated the performance of the registration using the dice similarity coefficient (DSC). In addition, to quantify the deformation regularity, we calculated the Jacobian determinant $det(D\phi^{-1})$ of the DDF. $det(D\phi^{-1})<0$ indicates the locations where folding has occurred. The proportion of folding voxels $\rho =\frac{\sum \delta (det(D\phi^{-1})<0)}{V}$ is computed to evaluate the topology-preserving performance [14].

We conducted experiments on the OASIS-3 dataset to evaluate the performance of the proposed fusion method and to compare it with three state-of-the state methods: AAW, MIScnn [15] and patchify [16]. Three kinds of deep learning models were trained: (1) VoxelMorph: this was a typical representative of unsupervised learning-based registration; (2) To produce a smoother DDF, a Jacobian constraint $loss_{JD}=0.5(\left|det(D\phi^{-1})\right|-det(D\phi^{-1}))$ was added to the loss function of VoxelMorph to reduce the folding of the DDF [2], denoted as VoxelMorph (JD); (3) Label-reg: this was a weakly-supervised image registration model [1].

### 3.3. Experimental Results

We used the trained VoxelMorph (JD) model to test the patches extracted from a sliding window with stride of 16. In our fusion method, Euclidean distance was chosen as the distance function. Table 2 shows the DSC values of CSF, GM, and WM, as well as the folding rate for the three fusion methods.

**Figure 8:**
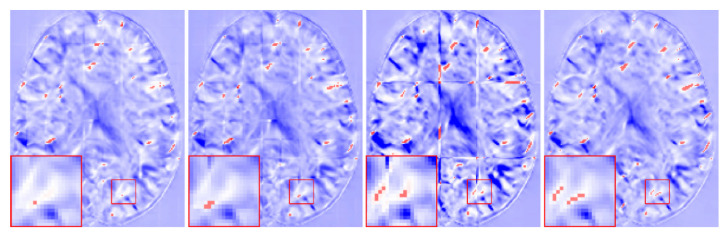
The fusion results for different methods. The positions of $\det(D\phi^{-1})<0$ are marked in red. From left to right: AAW method; MIScnn; patchify and our proposed.

As can be seen from Table 2, our method obtained higher DSC values when compared with the other three methods. However, the ρ values of MIScnn and AAW were lower, and the resulting DDF was smoother. In Figure 8, we show the DDFs of different fusion methods. It can be seen that the DDFs of AAW, MIScnn and patchify have obvious seam lines between patches, which indicates that the prediction of the edge region was inaccurate. Since our method considered the uncertainty of the edge, different weights were given to the center regions and the edge regions, which effectively eliminated the seams. From the enlarged red box, we can see that the DDF obtained by our method does not show grid-like artifacts. However, from the red markers, both our method and patchify retain many folding points which was mainly caused by the predicted model itself, while both the AAW and MIScnn methods changed the predicted values to some extent.

**Figure 9:**
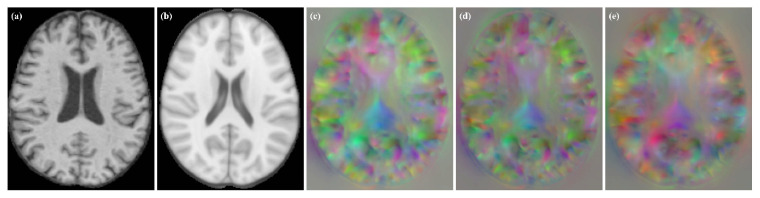
Moving image, fixed image and DDFs obtained for different models. (**a**) moving image; (**b**) fixed image; (**c**) VoxelMorph; (**d**) VoxelMorph (JD); (**e**) Label-reg.

To verify the robustness of the method, we tested it on VoxelMorph and Label-reg models. The experimental results are shown in Figure 9. We found that we obtained DDF without grid-like artifacts under different models.

### 3.4. Comparisons of the Results with Different Strides

In the AAW method, the stride has a great influence on the patch fusion process. Table 3 shows the DSC, ρ and fusion time of AAW and proposed methods with different strides.

**Figure 10:**
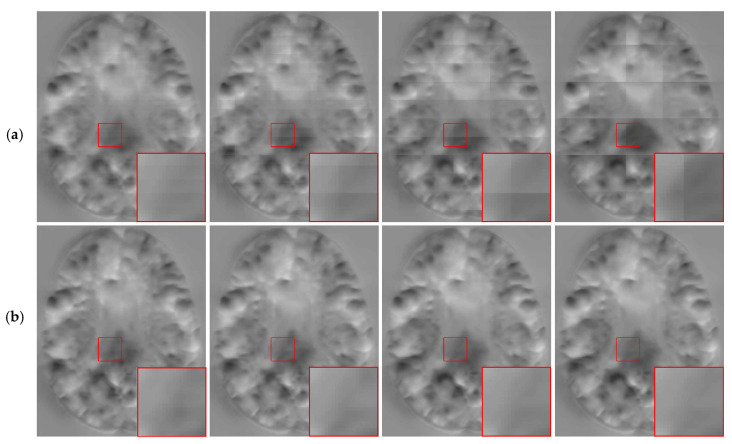
The results of different stride for AAW (**a**) and proposed method (**b**). The strides from left to right are 4 × 4 × 4, 8 × 8 × 8, 16 × 16 × 16 and 32 × 32 × 32.

In Table 3, when the stride decreases, the smoothness of DDF increases with a smaller ρ. However, the DSC of the CSF, GM and WM are nearly similar. As the number of patches increases with a small stride, the fusion time is significantly increased. Compared with the AAW method, the DSC and ρ values of our method are higher than those obtained by AAW under different strides. We found that the stride had little effect on the ρ obtained by our method, while the AAW method obtained a smoother deformation field with smaller strides. This further proves that our method can robustly maintain the predicted values of the registration model. In addition, the fusion times of the two methods has little difference. In Figure 10, we found that the DDFs of our method under different strides removed the grid-like artifacts, but the AAW method still has a small number of grid-like artifacts, even at a stride of 4 × 4 × 4. We can conclude that to obtain the DDF without grid-like artifacts, the AAW method requires a smaller stride while significantly increasing the fusion time. However, our method can obtain the DDF without grid-like artifacts in a shorter time, even under a larger stride, as well as a higher DSC value than that obtained by the AAW method.

### 3.5. Comparisons of the Results with Different Distance Functions

To explore the effect of distance functions on patch fusion, we choose Chebyshev distance, Manhattan distance, and Euclidean distance for our experiments. The results are shown in Table 4.

**Figure 11:**
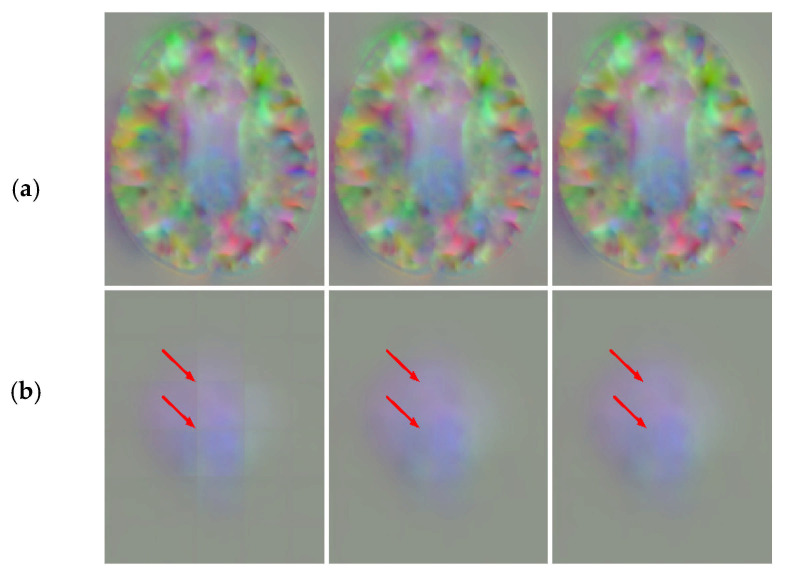
DDFs of three distance functions at 100 slices (**a**) and 160 slices (**b**) of the axial plane. The 1st column is the Chebyshev distance, the 2nd column is the Manhattan distance, and the 3rd column is the Euclidean distance.

From Table 4, it can be observed that the distance functions have little difference in the quantitative analysis. We also show the fusion results of the three distance functions in Figure 11. From the top row, we can see that the slices in the middle of the DDF introduced the three distance functions without grid-like artifacts. However, the Chebyshev distance still has a significant seam at the edge slice seen on the bottom row. In Figure 4, when using the Chebyshev distance as a distance function, the distances from the points at the edge to the centroid are equal. This gives the same weight to all predicted values at that point no matter how many times they overlap, which results in grid-like artifacts still appearing at the edge. Combined with Figure 5, the Chebyshev distance appears to undulate in the transition from low to high frequencies, which indicates that it is less stable at the edge and will retain some high-frequency information. Therefore, by our method, introducing the Euclidean distance or Manhattan distance can obtain the DDF without grid-like artifacts.

### 3.6. Comparisons of the Results with Different Weighting Methods

**Figure 12:**
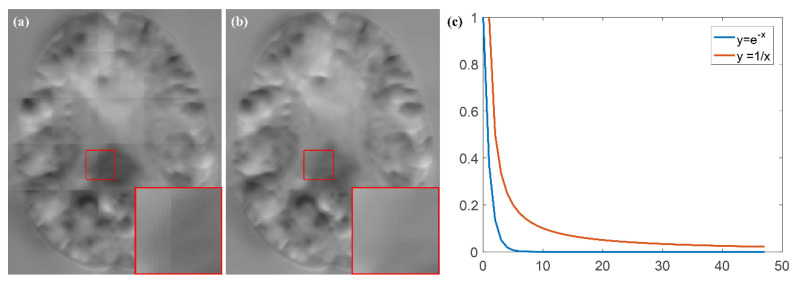
DDF results of different weighting methods and their corresponding function curves. (**a**) IDW; (**b**) proposed method; (**c**) weighting function curves.

The weighting method is the key to our fusion method. In this section, we chose the inverse-distance weighting (IDW) method to compare with our method. The distance function used is Euclidean distance. In Figure 12, we found that the IDW approach did not remove the grid-like artifacts. From the corresponding function curves in Figure 12c, it can be seen that in the central region (small distance), both weighting methods can give larger weighting coefficients, while in the edge region (larger distance), the IDW method produces larger weights than the EDW method. This indicates that the IDW method does not find the suitable weighting coefficients in the central and edge regions to eliminate the uncertainty of the edge, which results in its fusion results still having grid-like artifacts.

## 4. Discussion

Zero-padding is a relatively common operation in deep learning networks to keep the same input and output sizes. However, this operation is subject to uncertainty in edge prediction, which leads to grid-like artifacts in the patch fusion process. To solve this problem, we propose an exponential-distance-weighted fusion method. This method uses an exponential function to convert the distance from the predicted value of each patch to the center point into a set of weight coefficients. The larger the distance is, the smaller the weight coefficient is. Finally, the predicted DDF value of each voxel is obtained by a weighting method.

We performed experiments on the OASIS-3 dataset. By comparing our proposed method with AAW, MIScnn and patchify, three different fusion methods, our method obtained a seam-free DDF. Through a quantitative analysis, our overall DSC in the three types of brain tissue was 0.7784, and the other three methods were 0.7661, 0.7632, and 0.7668, respectively. It was found that our method is significantly better than these three methods. In addition, when compared with AAW and MIScnn, our fusion method did not change the predicted value of the registration model. To demonstrate the robustness and the effectiveness of our method, we also validated it on different models. In addition, we discussed the DDF results which were obtained under different strides, distance functions, and weighting methods. However, our method was found to have two shortcomings: (1) although our method can remove grid-like artifacts, it may change the true prediction results of the model after weighting; (2) the DDF obtained by our method is not smooth enough, and the folding rate still exists.

## 5. Conclusions

In this paper, we introduced a distance function to reduce grid-like artifacts when performing patch-based image registration. We demonstrated that our proposed EDW method has significant advantages over existing patch fusion methods. Moreover, our method is easy to implement into existing deep learning models, even if they are already trained. In the future, we will use the registration model to learn the weight coefficients of overlapping regions so that the contextual information can be fully considered, and the true prediction results of the model can be preserved. In addition, only the Euclidean distance, Manhattan distance, and Chebyshev distance were selected for experiments in this paper. Next, we will introduce more distance functions, and explore the influence of the distance function used, the network structure and the distance variance on this method.

## Figures and Tables

**Table 1:** Number of patches extracted in different strides during inference.

Stride	4 × 4 × 4	8 × 8 × 8	16 × 16 × 16	32 × 32 × 32
Number of Per Patch	20,625	2873	441	80

**Table 2:** Registration results of different fusion methods and the best results are shown in bold.

Method	DSC			ρ
	CSF	GM	WM	
AAW	0.7542	0.7253	0.8188	**0.0026**
MIScnn	0.7512	0.7223	0.8162	0.0028
Patchify	0.7573	0.7239	0.8192	0.0050
Proposed	**0.7621**	**0.7407**	**0.8325**	0.0042

Note: Arithmetic Average Weighted: AAW; Medical Image Segmentation with Convo-lutional Neural Networks: MIScnn.

**Table 3:** Registration results of different strides and the best results are shown in bold.

Stride	Method	DSC			ρ	Times (s)
		CSF	GM	WM		
4 × 4 × 4	AAW	0.7491	0.7231	0.8161	**0.0018**	**≈600**
	Proposed	**0.7578**	**0.7375**	**0.8293**	0.0037	≈1000
8 × 8 × 8	AAW	0.7512	0.7241	0.8173	**0.0022**	**91.3156**
	Proposed	**0.7593**	**0.7390**	**0.8307**	0.0040	97.0658
16 × 16 × 16	AAW	0.7542	0.7253	0.8188	**0.0026**	**14.1332**
	Proposed	**0.7621**	**0.7407**	**0.8325**	0.0042	14.7074
32 × 32 × 32	AAW	0.7556	0.7243	0.8183	**0.0034**	**2.6318**
	Proposed	**0.7627**	**0.7406**	**0.8326**	0.0042	2.7107

**Table 4:** Registration results of different distance functions and the best results are shown in bold.

Distance Function	DSC			ρ
	CSF	GM	WM	
Chebyshev	**0.7628**	0.7405	0.8326	0.0042
Manhattan	0.7626	**0.7406**	0.8326	0.0042
Euclidean	0.7627	**0.7406**	0.8326	0.0042

## Data Availability

In this work, the sample data are all derived from the Open Access Series of Imaging Studies (OASIS) database, https://www.oasis-brains.org, accessed on 15 July 2021.

## References

[1] Hu Y., Modat M., Gibson E., Li W., Ghavami N., Bonmati E., Wang G., Bandula S., Moore M., Emberton C. (2018). Weakly-supervised convolutional neural networks for multimodal image registration. Med. Image Anal..

[2] Hering A., Häger S., Moltz J., Lessmann N., Heldmann S., van Ginneken B. (2021). CNN-based Lung CT Registration with Multiple Anatomical Constraints. Med. Image Anal..

[3] Balakrishnan G., Zhao A., Sabuncu M.R., Guttag J., Dalca A.V. (2019). Voxelmorph: A learning framework for deformable medical image registration. IEEE Trans. Med. Imaging.

[4] Fu Y., Lei Y., Wang T., Curran W.J., Liu T., Yang X. (2020). Deep learning in medical image registration: A review. Phys. Med. Biol..

[5] Yang X., Kwitt R., Styner M., Niethammer M. (2017). Quicksilver: Fast predictive image registration–a deep learning approach. NeuroImage.

[6] Cao X., Yang J., Zhang J., Wang Q., Yap P.T., Shen D. (2018). Deformable image registration using a cue-aware deep regression network. IEEE Trans. Med. Imaging.

[7] Fan J., Cao X., Yap P.T., Shen D. (2019). BIRNet: Brain image registration using dual-supervised fully convolutional networks. Med. Image Anal..

[8] Hu S., Zhang L., Li G., Liu M., Fu D., Zhang W. (2019). Brain Deformable Registration Using Global and Local Label-Driven Deep Regression Learning in the First Year of Life. IEEE Access.

[9] Malkauthekar M.D. Analysis of Euclidean distance and Manhattan distance measure in Face recognition. Proceedings of the Third International Conference on Computational Intelligence and Information Technology (CIIT 2013).

[10] Coghetto R. (2016). Chebyshev distance. Formaliz. Math..

[11] Wang L., Xie C., Lin Y., Zhou H., Chen K., Cheng D., Dubost F., Collery B., Khanal B., Khanal B. (2021). Evaluation and Comparison of Accurate Automated Spinal Curvature Estimation Algorithms with Spinal Anterior-posterior X-Ray Images: The AASCE2019 Challenge. Med. Image Anal..

[12] Ma X., Xi B., Zhang Y., Zhu L., Sui X., Tian G., Yang J.A. (2020). Machine Learning-based Diagnosis of Thyroid Cancer Using Thyroid Nodules Ultrasound Images. Curr. Bioinform..

[13] LaMontagne P.J., Benzinger T.L., Morris J.C., Keefe S., Hornbeck R., Xiong C., Grant E., Hassenstab J., Moulder K.G., Vlassenko A. (2019). OASIS-3: Longitudinal neuroimaging, clinical, and cognitive dataset for normal aging and Alzheimer disease. MedRxiv.

[14] Zhang S., Liu P.X., Zheng M., Shi W. (2020). A diffeomorphic unsupervised method for deformable soft tissue image registration. Comput. Biol. Med..

[15] Müller D., Kramer F. (2021). MIScnn: A framework for medical image segmentation with convolutional neural networks and deep learning. BMC Med. Imaging.

[16] Wu W. Patchify. https://github.com/dovahcrow/patchify.py.

